# Supplementary material for: Three million images and morphological profiles of cells treated with matched chemical and genetic perturbations
Source: Nat Methods. 2024 Apr 9;21(6):1114–21. doi: 10.1038/s41592-024-02241-6 (PMC11166567; doi:10.1038/s41592-024-02241-6)
Supplement: Supplementary file 1 — Supplementary Tables 1–10. [file 41592_2024_2241_MOESM1_ESM.pdf]

nature methods

Resource

https://doi.org/10.1038/s41592-024-02241-6

Three million images and morphological profiles of cells treated with matched chemical and genetic perturbations

In the format provided by the authors and unedited

Perturbation modality	Time point – short	Time point – long
Compound	24-hour	48-hour
ORF	48-hour	96-hour
CRISPR	96-hour	144-hour

Supplementary Table 1: **Perturbation time points**. Description of the *short* and *long* time points for each perturbation modality.

Type	Description	n
Replicate retrieval	compound_long_A549	306
Replicate retrieval	compound_long_U2OS	306
Replicate retrieval	compound_short_A549	306
Replicate retrieval	compound_short_U2OS	306
Replicate retrieval	crispr_long_A549	305
Replicate retrieval	crispr_long_U2OS	305
Replicate retrieval	crispr_short_A549	305
Replicate retrieval	crispr_short_U2OS	305
Replicate retrieval	orf_long_A549	160
Replicate retrieval	orf_long_U2OS	160
Replicate retrieval	orf_short_A549	160
Replicate retrieval	orf_short_U2OS	160
Sister perturbation retrieval	compound_long_A549	402
Sister perturbation retrieval	compound_long_U2OS	285
Sister perturbation retrieval	compound_short_A549	315
Sister perturbation retrieval	compound_short_U2OS	300
Sister perturbation retrieval	crispr_long_A549	73
Sister perturbation retrieval	crispr_long_U2OS	42
Sister perturbation retrieval	crispr_short_A549	73
Sister perturbation retrieval	crispr_short_U2OS	73
Gene compound retrieval	compound_long_crispr_long_A549	134
Gene compound retrieval	compound_long_crispr_long_U2OS	92
Gene compound retrieval	compound_long_crispr_short_A549	135
Gene compound retrieval	compound_long_crispr_short_U2OS	115
Gene compound retrieval	compound_long_orf_long_A549	8
Gene compound retrieval	compound_long_orf_long_U2OS	29
Gene compound retrieval	compound_long_orf_short_A549	8
Gene compound retrieval	compound_long_orf_short_U2OS	53

Gene compound retrieval	compound_short_crispr_long_A549	128
Gene compound retrieval	compound_short_crispr_long_U2OS	102
Gene compound retrieval	compound_short_crispr_short_A549	130
Gene compound retrieval	compound_short_crispr_short_U2OS	125
Gene compound retrieval	compound_short_orf_long_A549	8
Gene compound retrieval	compound_short_orf_long_U2OS	30
Gene compound retrieval	compound_short_orf_short_A549	8
Gene compound retrieval	compound_short_orf_short_U2OS	58

Supplementary Table 2: n = Number of biologically independent samples (cells treated with compounds, CRISPR or ORF reagents) in each box plot in Figure 4.

**Cell**	**Fisher's exact statistic**	**p value**
U2OS	2.64156	9.40265e-23
A549	2.19389	6.52766e-15

Supplementary Table 3: **Comparing the directionality of ORF-CRISPR cosine similarities.** We performed one-sided Fisher's exact test to determine whether the cosine similarities between ORFs and CRISPR guides were positive or negative. ORF-CRISPR pairs that were beyond the 5th and 95th percentile of the null distribution (cosine similarities of ORF and CRISPR guides targeting different genes) were used in the Fisher's test. A positive, significant ($p < 0.05$) statistic means that the ORF-CRISPR cosine similarities are more positive than negative.

**Cell**	**Genetic Perturbation**	**Fisher's exact statistic**	**p value**
A549	CRISPR	2.03487	4.50572e-07
A549	ORF	2.19404	6.53061e-08
U2OS	CRISPR	2.81489	6.53061e-08
U2OS	ORF	2.63868	4.51155e-12

Supplementary Table 4: **Comparing the directionality of Compound-CRISPR and Compound-ORF cosine similarities.** We performed one-sided Fisher's test to determine whether the cosine similarities between compounds and each genetic perturbation type (ORF and CRISPR guides) were positive or negative. Compound-genetic perturbation pairs that were beyond the 5th and 95th percentile of the null distribution (cosine similarities of compound-genetic perturbations targeting different proteins encoded by given genes) were used in the Fisher's test. A positive, significant ($p < 0.05$) statistic means that the compound-genetic perturbation cosine similarities are more positive than negative.

**Cell**	**Genetic**	**pert_iname**	**Metadata_**	**moa_list**	**Cosine**

	Perturbation		matching_ target		similarity
U2OS	CRISPR	BI-2536	PLK1	PLK inhibitor	0.779615
U2OS	CRISPR	AMG900	AURKB	Aurora kinase inhibitor	0.72932
U2OS	CRISPR	danusertib	AURKB	Aurora kinase inhibitor, growth factor receptor inhibitor	0.654305
U2OS	ORF	PP-2	ABL1	src inhibitor	0.643488
U2OS	ORF	ponatinib	LYN	Bcr-Abl kinase inhibitor, FLT3 inhibitor, PDGFR tyrosine kinase receptor inhibitor	0.61835
U2OS	ORF	BI-2536	BRD4	PLK inhibitor	0.598121
U2OS	ORF	pentostatin	ADA	adenosine deaminase inhibitor, ribonucleotide reductase inhibitor	0.583311
U2OS	ORF	hexestrol	AKR1C1	synthetic estrogen	0.5711
U2OS	ORF	pyrrolidine-dithiocarbamate	HSD11B1	NFkB pathway inhibitor	0.566448
U2OS	CRISPR	sodium-butyrate	FFAR2	HDAC inhibitor	0.560779
U2OS	CRISPR	TG-003	CLK1	CLK inhibitor	-0.326471
U2OS	CRISPR	GSK3787	PPARD	PPAR receptor antagonist	-0.338408
U2OS	ORF	amlexanox	FGF1	histamine receptor modulator	-0.343726
U2OS	ORF	pazopanib	FGF1	KIT inhibitor, PDGFR tyrosine kinase receptor inhibitor, VEGFR inhibitor	-0.354573
U2OS	CRISPR	PF-06463922	ALK	ALK tyrosine kinase receptor inhibitor	-0.372329
U2OS	ORF	sotrastaurin	PRKCE	PKC inhibitor	-0.383463
U2OS	CRISPR	benzamil	ASIC1	sodium channel blocker	-0.424186
U2OS	CRISPR	ZM-336372	LCK	RAF inhibitor	-0.44488
U2OS	ORF	GSK2110183	AKT1	AKT inhibitor	-0.481628
U2OS	ORF	ZM-336372	MAPK14	RAF inhibitor	-0.547655

Supplementary Table 5: **Compound-genetic perturbation similarity in U2OS cells**. The top 10 positively correlated and negatively correlated compound-genetic perturbations pairs in U2OS. pert_iname is the name of the compound, Metadata_matching_target refers to the target of the compound and genetic perturbation and moa_list is the list of mechanism of action annotation(s) for each compound.

Cell	Genetic Perturbation	pert_iname	Metadata_matching_target	moa_list	Cosine similarity
A549	CRISPR	BI-2536	PLK1	PLK inhibitor	0.757815
A549	CRISPR	AMG900	AURKB	Aurora kinase inhibitor	0.729485
A549	CRISPR	NSC-663284	CDC25A	CDC inhibitor	0.720625
A549	CRISPR	KI-16425	LPAR1	lysophosphatidic acid receptor antagonist	0.680091
A549	CRISPR	danusertib	AURKB	Aurora kinase inhibitor, growth factor receptor inhibitor	0.621753
A549	CRISPR	fludarabine-phosphate	DCK	ribonucleotide reductase inhibitor	0.618721
A549	CRISPR	GSK1070916	AURKB	Aurora kinase inhibitor	0.58189
A549	CRISPR	bepridil	TNNC1	calcium channel blocker	0.557164
A549	CRISPR	carzenide	CA14		0.55219
A549	CRISPR	aminopurvalanol-a	CDK2	CDK inhibitor, tyrosine kinase inhibitor	0.534492
A549	ORF	pazopanib	FGF1	KIT inhibitor, PDGFR tyrosine kinase receptor inhibitor, VEGFR inhibitor	-0.35889
A549	CRISPR	citric-acid	AKR1B1	coagulation factor inhibitor	-0.36625
A549	ORF	ibutilide	KCNH7	potassium channel blocker	-0.369209
A549	CRISPR	JTE-607	TNF	cytokine production inhibitor	-0.377579
A549	CRISPR	salicylic-acid	AKR1C1	cyclooxygenase inhibitor	-0.391766
A549	CRISPR	epoprostenol	PTGIS	prostacyclin analog	-0.395514
A549	CRISPR	rifamycin	SLCO2B1	DNA directed RNA polymerase inhibitor	-0.405275
A549	ORF	sulfasalazine	SLC7A11	cyclooxygenase inhibitor	-0.417641
A549	CRISPR	ibutilide	CACNG1	potassium channel blocker	-0.492405
A549	CRISPR	sorbinil	AKR1B1	aldose reductase inhibitor	-0.541175

Supplementary Table 6: **Compound-genetic perturbation similarity in A549 cells**. The top 10 positively correlated and negatively correlated compound-genetic perturbations pairs in A549. pert_iname is the name of the compound, Metadata_matching_target refers to the target of the compound and genetic perturbation and moa_list is the list of mechanism of action annotation(s) for each compound.

Number of compounds (N)	Number of proteins targeted by N compounds

2	83
3	34
4	19
5	8
1	6
6	4
8	2
10	2
7	1
9	1

Supplementary Table 7: **Number of genes' products targeted by a given number of compounds.** Most genes' products (proteins) are targeted by two compounds, though some proteins encoded by given genes are targeted by as many as ten compounds because many compounds in the set are annotated as having multiple protein targets.

Number of protein targets (N)	Number of compounds targeting N protein targets
1	218
2	49
3	23
4	7
5	4
6	3
7	1
8	1

Supplementary Table 8: **Number of compounds with a given number of protein targets**. Most compounds (n=218) are annotated as targeting a single gene's product, but there are sixteen compounds that target four or more proteins encoded by given genes. Because annotations are incomplete, there may be more targets per compound than noted here.

Number of grouped protein targets (N)	Number of compounds targeting N protein targets
1	241

2	37
3	20
4	3
5	2
6	2
8	1

Supplementary Table 9: **Number of compounds with a given number of protein targets, grouped by gene product family.** When members of the same gene family are grouped together for this analysis, for example NTRK1, NTRK2 and NTRK3, then only eight compounds target four or more genes' products or gene product families; the majority of compounds (241) target only a single gene product or gene product family.

Number of associated gene targets (N)	Number of gene families with N associated gene targets in the final list
1	92
2	16
3	2

Supplementary Table 10: **Number of gene families with a given number of associated gene targets.** Closely related genes are called *families.* Gene family names were downloaded from https://www.genenames.org/download/custom/. To maximize the diversity of genes and reduce the frequency of retrieving a gene's family member as an "incorrect" match for a given compound, the 130 genes chosen for the experiment belonged to 110 diverse gene families. The additional 30 associated genes in the experiment were targets of the positive control compounds in the experiment.
