# Supplementary material for: Three million images and morphological profiles of cells treated with matched chemical and genetic perturbations
Source: Nat Methods. 2024 Apr 9;21(6):1114–21. doi: 10.1038/s41592-024-02241-6 (PMC11166567; doi:10.1038/s41592-024-02241-6)
Supplement: Supplementary file 2 — Reporting Summary [file 41592_2024_2241_MOESM2_ESM.pdf]

nature portfolio

Corresponding author(s): A.E. Carpenter

Last updated by author(s): Feb 21, 2024

Reporting Summary

Nature Portfolio wishes to improve the reproducibility of the work that we publish. This form provides structure for consistency and transparency in reporting. For further information on Nature Portfolio policies, see our Editorial Policies and the Editorial Policy Checklist.

Statistics

For all statistical analyses, confirm that the following items are present in the figure legend, table legend, main text, or Methods section.

n/a	Confirmed	
☐	☒	The exact sample size (*n*) for each experimental group/condition, given as a discrete number and unit of measurement
☐	☒	A statement on whether measurements were taken from distinct samples or whether the same sample was measured repeatedly
☐	☒	The statistical test(s) used AND whether they are one- or two-sided<br>*Only common tests should be described solely by name; describe more complex techniques in the Methods section.*
☐	☒	A description of all covariates tested
☐	☒	A description of any assumptions or corrections, such as tests of normality and adjustment for multiple comparisons
☐	☒	A full description of the statistical parameters including central tendency (e.g. means) or other basic estimates (e.g. regression coefficient) AND variation (e.g. standard deviation) or associated estimates of uncertainty (e.g. confidence intervals)
☐	☒	For null hypothesis testing, the test statistic (e.g. *F*, *t*, *r*) with confidence intervals, effect sizes, degrees of freedom and *P* value noted<br>*Give P values as exact values whenever suitable.*
☒	☐	For Bayesian analysis, information on the choice of priors and Markov chain Monte Carlo settings
☒	☐	For hierarchical and complex designs, identification of the appropriate level for tests and full reporting of outcomes
☐	☒	Estimates of effect sizes (e.g. Cohen's *d*, Pearson's *r*), indicating how they were calculated

Our web collection on statistics for biologists contains articles on many of the points above.

Software and code

Policy information about availability of computer code

Data collection	No software was used to collect data.
Data analysis	Data analysis was performed using python code written in a Jupyter notebook environment. Python libraries used for data analysis include Numpy, Scipy, scikit-learn and pandas. Plots were generated using matplotlib, seaborn and Plotly libraries. Fiji7's Magic Montage plugin, Lucidchart and inkscape were used for generating montages, creating schematics and for adding text to images. Two-dimensional representations of image-based profiles in Figs. 2 and Extended Data Figs. 5-10 were generated using UMAP . The code used in this study is available at https://github.com/jump-cellpainting/2024_Chandrasekaran_NatureMethods. It is available for use under the BSD 3-clause license, a permissive open-source license.

For manuscripts utilizing custom algorithms or software that are central to the research but not yet described in published literature, software must be made available to editors and reviewers. We strongly encourage code deposition in a community repository (e.g. GitHub). See the Nature Portfolio guidelines for submitting code & software for further information.

March 2021

Data

Policy information about availability of data

All manuscripts must include a data availability statement. This statement should provide the following information, where applicable:

- Accession codes, unique identifiers, or web links for publicly available datasets
- A description of any restrictions on data availability
- For clinical datasets or third party data, please ensure that the statement adheres to our policy

Well-level morphological profiles, image analysis pipelines, profile generation pipelines, plate maps and plate and compound metadata, and instructions for retrieving the cell images and single cell profiles are publicly available online at https://broad.io/cpjump1.

Cell Painting images and single-cell profiles are available at the Cell Painting Gallery on the Registry of Open Data on AWS (https://registry.opendata.aws/cellpainting-gallery/) under accession number cpg0000-jump-pilot. For well-level aggregated profiles, we use GitHub as the hosting platform and the files are stored in GitLFS.

We have released the data with a CC0 license.

Human research participants

Policy information about studies involving human research participants and Sex and Gender in Research.

Reporting on sex and gender	Not applicable
Population characteristics	Not applicable
Recruitment	Not applicable
Ethics oversight	Not applicable

Note that full information on the approval of the study protocol must also be provided in the manuscript.

Field-specific reporting

Please select the one below that is the best fit for your research. If you are not sure, read the appropriate sections before making your selection.

☒ Life sciences ☐ Behavioural & social sciences ☐ Ecological, evolutionary & environmental sciences

For a reference copy of the document with all sections, see nature.com/documents/nr-reporting-summary-flat.pdf

Life sciences study design

All studies must disclose on these points even when the disclosure is negative.

Sample size

Experiments involved cultured cells where the number of cells is governed by the size of the growth vessel; The number of replicates for various biological experiments were chosen based on each expert's knowledge of the variability in the type of experiment. We found from previous experiments that the quality of signal in the data did not improve significantly beyond four replicates.

Data exclusions

No data was excluded from the analyses.

Replication

All data presented indicates the number of replicates represented. Experiment was performed with four replicates of each treatment for each experimental condition. All replications were successful.

Randomization

We designed the experimental plates such that genes that are perturbed by CRISPR and ORF reagents, their products, are always targeted by two compounds. We randomized the location of each sample on the experimental plate as long as they satisfied the following criteria:

- Both of the compounds that have the same target will either be in the inner wells or in the outer wells. They will not be split such that one of the compounds is in the inner well while the other is in the outer well. where the outer wells are the two rows and columns closest to the edge of the plate and the inner wells are the rest of the wells on the plate.
- The gene associated with the target of outer well compounds will be in the outer wells of the genetic perturbation plate.
- All the positive control compounds are in the inner wells.

Blinding

Experiments involved cells; group allocation is not applicable.

Reporting for specific materials, systems and methods

We require information from authors about some types of materials, experimental systems and methods used in many studies. Here, indicate whether each material, system or method listed is relevant to your study. If you are not sure if a list item applies to your research, read the appropriate section before selecting a response.

Materials & experimental systems

n/a	Involved in the study
☒	☐ Antibodies
☐	☒ Eukaryotic cell lines
☒	☐ Palaeontology and archaeology
☒	☐ Animals and other organisms
☒	☐ Clinical data
☒	☐ Dual use research of concern

Methods

n/a	Involved in the study
☒	☐ ChIP-seq
☒	☐ Flow cytometry
☒	☐ MRI-based neuroimaging

Eukaryotic cell lines

Policy information about cell lines and Sex and Gender in Research

Cell line source(s)	A549<br>Catalog: CCL-185<br>Lot: 70018877<br><br>U2OS<br>Catalog: HTB-96<br>Lot: 70016635
Authentication	Since the cells were ordered directly from ATCC, we did not authenticate via SNP or STR profiling.
Mycoplasma contamination	We tested the parental cells ordered from ATCC for mycoplasma contamination a few months into culturing them to ensure they remained negative.
Commonly misidentified lines<br>(See ICLAC register)	To our knowledge neither of these cell lines, U-2 OS nor A549 are commonly misidentified.
